# Subacute Zinc Administration and L-NAME Caused an Increase of NO, Zinc, Lipoperoxidation, and Caspase-3 during a Cerebral Hypoxia-Ischemia Process in the Rat

**DOI:** 10.1155/2013/240560

**Published:** 2013-08-07

**Authors:** Victor Manuel Blanco-Alvarez, Patricia Lopez-Moreno, Guadalupe Soto-Rodriguez, Daniel Martinez-Fong, Hector Rubio, Juan Antonio Gonzalez-Barrios, Celia Piña-Leyva, Maricela Torres-Soto, María de Jesus Gomez-Villalobos, Daniel Hernandez-Baltazar, Eduardo Brambila, José Ramon Eguibar, Araceli Ugarte, Jorge Cebada, Bertha Alicia Leon-Chavez

**Affiliations:** ^1^Facultad de Ciencias Químicas, Benemérita Universidad Autónoma de Puebla, 14 sur y Avenida San Claudio Edif. 105A, CU, Col. San Manuel, 72570 Puebla, PUE, Mexico; ^2^Departamento de Fisiología, Biofísica y Neurociencias, CINVESTAV, Apartado Postal 14-740, 07000 México, DF, Mexico; ^3^Facultad de Medicina, Universidad Autónoma de Yucatán, Av. Itzáez No. 498 x 59 y 59-A Col. Centro, C. P. 97000 Mérida, YUC, Mexico; ^4^Laboratorio de Medicina Genómica del Hospital Regional “1° de Octubre”, ISSSTE, Avenida Instituto Politécnico Nacional 1669, 07760 México, DF, Mexico; ^5^Instituto de Fisiología, Benemérita Universidad Autónoma de Puebla, 14 sur 6301, San Claudio, 72570 Puebla, PUE, Mexico; ^6^Escuela de Biología, Benemérita Universidad Autónoma de Puebla, Blvd. Valsequillo y Avenida San Claudio Edif. 76, CU, Col. San Manuel, 72570 Puebla, PUE, Mexico

## Abstract

Zinc or L-NAME administration has been shown to be protector agents, decreasing oxidative stress and cell death. However, the treatment with zinc and L-NAME by intraperitoneal injection has not been studied. The aim of our work was to study the effect of zinc and L-NAME administration on nitrosative stress and cell death. Male Wistar rats were treated with ZnCl_2_ (2.5 mg/kg each 24 h, for 4 days) and N-**ω**-nitro-L-arginine-methyl ester (L-NAME, 10 mg/kg) on the day 5 (1 hour before a common carotid-artery occlusion (CCAO)). The temporoparietal cortex and hippocampus were dissected, and zinc, nitrites, and lipoperoxidation were assayed at different times. Cell death was assayed by histopathology using hematoxylin-eosin staining and caspase-3 active by immunostaining. The subacute administration of zinc before CCAO decreases the levels of zinc, nitrites, lipoperoxidation, and cell death in the late phase of the ischemia. L-NAME administration in the rats treated with zinc showed an increase of zinc levels in the early phase and increase of zinc, nitrites, and lipoperoxidation levels, cell death by necrosis, and the apoptosis in the late phase. These results suggest that the use of these two therapeutic strategies increased the injury caused by the CCAO, unlike the alone administration of zinc.

## 1. Introduction

A stroke causes disability and death. Extensive studies have shown the participation of oxidative stress as the mechanism underlying a cerebral ischemia injury. Tissue plasminogen activator (tPA) has been the only drug approved by the FDA for treating ischemic stroke, but its use is restricted because to its adverse effects [1]. There is evidence that zinc is a cytoprotector agent [2–4], increasing the antioxidant capacity and decreasing the iron-catalyzed lipid peroxidation, as well as the apoptosis [5].

Zinc has a dual role during the pathological process of stroke. The accumulation of zinc has cytotoxic properties [6–12]. However, the administration of zinc protoporphyrin, zinc ion, or protoporphyrin decreases the focal cerebral ischemia [4] and prevents neuron death [2, 3]. The beneficial action of zinc is caused by its antioxidant properties. The zinc treatment prevents lipid peroxidation and increases glutathione availability in Wilson's disease [13]. Zinc decreases the apoptosis through inhibition of Bax and Bak activation and cytochrome c release [14]. In addition, zinc is a potent inhibitor of the apoptotic proteases, caspase-3 [15, 16], and caspase-8 [17]. 

Studies have shown the participation of nitric oxide (NO) in zinc accumulation, the increase of cleaved caspase-3 and lipoperoxidation during a process of cerebral hypoxia-ischemia [18], and through release of zinc from presynaptic buttons [19]. Nitric oxide causes the release of zinc from metallothionein by destroying zinc-sulphur clusters without concomitant formation of S-nitrosothiol [20]. However, NO plays a critical role in the protection of the liver from oxidative stress. The mechanisms involved include its role as an antioxidant agent of iron that decreases the oxidative stress in rat hepatocytes [21] or through the pathway of Akt-eNOS-NO-HIF in ischemia postconditioning [22]. The NO has protective properties on the brain during an acute ischemic stroke, but the increase of the NOS activity causes the alteration of microvasculature integrity and edema formation during cerebral ischemia-reperfusion injuries in the rat, without changing arterial blood pressure or blood flow in the ischemic regions [23]. There is evidence that the inhibition of NO by N-*ω*-nitro-L-arginine methyl ester (L-NAME) decreases the zinc levels and increases cardiac-necrosis marker levels detected in the plasma of rabbits [24]. However, L-NAME administration decreases cell death after the ischemia [18].

These antecedents support the idea that NO and zinc have a dual role during the ischemia process. However, the coadministration of zinc and an NOS inhibitor has not been defined. In this work we study the prophylactic effects of the subacute administration of zinc (2.5 mg/kg, each 24 h for 4 days) and L-NAME (10 mg/kg, one hour before a common carotid-artery occlusion (CCAO)) on nitrite levels, and the production of malonyldialdehyde (MDA) + 4-hydroxialkenals (HAE) at different hours pre- and postreperfusion. Histopathological changes were evaluated through immunoreactivity against cleaved caspase-3 and hematoxylin-eosin staining. Our results support the idea that NO in the early phase is a cytoprotector agent and NO in the later phase acts as a cytotoxic agent. Zinc administration alone has a cytoprotector role against the damage caused by CCAO, but the coadministration of zinc and L-NAME causes more damage compared to the hypoxia-ischemia process.

## 2. Materials and Methods

### 2.1. Experimental Animals

Male Wistar rats between 190 g and 240 g were obtained from the vivarium of the CINVESTAV. The animals were maintained in adequate animal rooms with controlled conditions of temperature (22 ± 1°C) and a light-dark cycle (12 h-12 h light-dark; light onset at 0700). Food and water were provided ad libitum. All procedures were in accordance with the Mexican current legislation, the NOM-062-ZOO-1999 (SAGARPA), based on the Guide for the Care and Use of Laboratory Animals, NRC. The Institutional Animal Care and Use Committee (IACUC) approved our animal-use procedures with the protocol number 09-102. All efforts were made to minimize animal suffering.

### 2.2. Zinc and L-NAME Administration

 The rats were grouped into different treatments: (1) control (without treatment), (2) CZn96h; control treated with ZnCl_2_ (2.5 mg/kg each 24 h for 4 days), from which the brain was obtained at 24 h, 48 h, 72 h, and 96 h postadministration, (3) Zn96h + CCAO; rats treated with a subacute administration of zinc and transient ischemia through a common carotid artery occlusion (CCAO), which was caused for 10 min; the brain was obtained at different hours (4 h, 8 h, 12 h, 24 h, 36 h, 72 h, 96 h, and 168 h postreperfusion), (4) Zn96h + L-NAME control; the rats received a subacute administration of zinc for 4 days plus L-NAME (10 mg/kg), and (5) Zn96h + L-NAME + CCAO; these rats received all treatments, and the brain was obtained at different hours (4 h, 8 h, 12 h, 24 h, 36 h, 72 h, 96 h, and 168 h postreperfusion).

### 2.3. Nitric Oxide Determination

 The temporoparietal cortex and hippocampus of all studied groups (*n* = 5 in each group) were mechanically homogenized in phosphate-buffered saline solution (PBS), pH 7.4, and centrifuged at 12,500 rpm for 30 min at 4°C by using a 17TR microcentrifuge (Hanil Science Industrial Co. Ltd; Inchun, Korea). The NO production was assessed by the accumulation of nitrites (NO_2_^−^) in the supernatants of homogenates, as described elsewhere [18, 25]. Briefly, the nitrite concentration in 100 *μ*L of supernatant was measured by using a colorimetric reaction generated by the addition of 100 *μ*L of Griess reagent, which was composed of equal volumes of 0.1% N-(1-naphthyl) ethylenediamine dihydrochloride and 1.32% sulfanilamide in 60% acetic acid. The absorbance of the samples was determined at 540 nm with a SmartSpec 3000 spectrophotometer (Bio-Rad; Hercules, CA, USA) and interpolated by using a standard curve of NaNO_2_ (1 to 10 *μ*M) to calculate the nitrite content. 

### 2.4. Measure of Blood Pressure

 The blood pressure was measured in all studied groups (*n* = 5 in each group). Systolic and diastolic blood pressures were measured using the tail-cuff method by using the XBP1000 Blood Pressure System from Kent Scientific Corporation. Systolic and diastolic blood pressures (mean ± SE, mm Hg) were measured in all animals 24-h before and after each treatment as previously described [26].

### 2.5. Lipoperoxidation

Malonyldialdehyde (MDA) and 4-hydroxyalkanal (HEA) were measured in supernatants of homogenates of the temporoparietal cortex and hippocampus using the method described elsewhere [18, 25]. The colorimetric reaction in 200 *μ*L of the supernatant was produced by the subsequent addition of 0.650 mL of 10.3 mM N-methyl-2phenyl-indole diluted in a mixture of acetonitrile : methanol (3 : 1). The reaction was started by the addition of 150 *μ*L of methanesulfonic acid. The reaction mixture was strongly vortexed and incubated at 45°C for 1 h and then centrifuged at 3000 rpm for 10 min. The absorbance in the supernatant was read at 586 nm with a SmartSpec 3000 spectrophotometer (Bio-Rad; Hercules, CA, USA). The absorbance values were compared to a standard curve in the concentration range of 0.5 to 5 *μ*M of 1,1,3,3-tetramethoxypropane (10 mM stock) to calculate the malonyldialdehyde and 4-hydroxyalkanal contents in the samples.

### 2.6. Immunolabeling of Cleaved Caspase-3

The immunoreactivity against cleaved caspase-3 was analyzed by an immunohistochemical method [25]. The fixed brains with 4% paraformaldehyde in PBS were maintained overnight in PBS containing 30% sucrose at 4°C. Then, each brain was frozen and sectioned into 10 *μ*m slices on the coronal plane using a Leica SM100 cryostat (Leica Microsystems, Nussloch, Germany). Slices were individually collected in a 24-well plate containing PBS and used for the immunohistochemistry for cleaved caspase-3. The slices were incubated with PBS-Triton (0.1%) and later with 10% horse serum in PBS-Triton (0.1%) for 60 min at room temperature. The slices were incubated overnight with a rabbit polyclonal antibody against cleaved caspase-3 (1 : 300 dilution; Cell Signaling Technology, Danvers, MA, USA) and then with a 1 : 600 dilution of the secondary biotinylated goat antibody anti-rabbit IgG (H + L) (Vector Laboratories, Burlingame, CA, USA) for 2 hours at room temperature. After rinsing, the slices were incubated with streptavidin-horseradish peroxidase conjugate (BRL Inc., Gaithersburg, MD, USA) and diluted 1 : 400, again for 30 minutes at room temperature. The peroxidase reaction was developed by immersion in a freshly prepared solution of 0.02% 3,3′-diaminobenzidine (DAB, Sigma). The slices were counterstained with cresyl violet. The caspase-3 immunoreactivity was analyzed with a magnification of 5x, 20x, and 40x using a Leica DMIRE2 microscope (Leica Microsystems, Wetzlar, Germany). The images were digitalized with a Leica DC300F camera (Leica Microsystems, Nussloch, Germany) and analyzed with workstation Leica FW4000, version V1.2.1 (Leica Microsystems Vertrieb GmbH; Bensheim, Germany).

The histopathology study of the temporoparietal cortex and hippocampus from the brains of each experimental group was analyzed in coronary brain slices by hematoxylin-eosin staining at 24 h and 7-day postreperfusion (*n* = 3 in each group). The 3 *μ*m paraffin-embedded tissue sections were stained with hematoxylin and eosin and examined at a magnification objective of 40x (Mod BM 1000 Leica, Jenopika Camera, Wetzlar, Germany). Digital micrographs were made from 5 randomly selected fields of each tissue section of each experimental group (Progress capture pro 2.1, Leica). 

### 2.7. Statistical Analysis

All values are the mean ± SE obtained from at least 5 independent experiments. The significance of differences was analyzed by an ANOVA test and a post-hoc Dunnet test to compare with control group and an unpaired Student's *t*-test to compare between groups. The data were analyzed using the Graph Pad Prism software (version 5.00). A significant value was considered at *P* < 0.05.

## 3. Results

**Figure 1:**
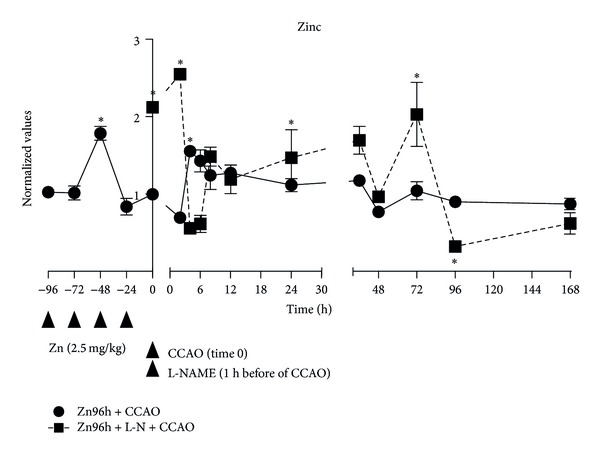
Effect of subacute administration of zinc and L-NAME on zinc levels during a cerebral hypoxia-ischemic process. The zinc levels were assayed by the Johnson method described elsewhere [18, 25]. Each value is the mean ± SE of 5 independent experiments made in triplicate. Zn96h + CCAO: preventive subacute administration of zinc (2.5 mg/kg intraperitoneal each 24 hours during 4 days) and common carotid-artery occlusion (CCAO) for 10 min. Zn96h + L-NM + CCAO: rats treated with zinc in the presence of an inhibitor of nitric oxide synthase (L-NAME) one hour before the CCAO. *P < 0.05, an ANOVA test and a post-hoc Dunnet test to compare with the control group, and ^†^ *P* < 0.05, unpaired Student's *t*-test to compare between groups.

The zinc levels were determined in homogenized supernatants of the temporoparietal cortex at different hours, before and after the CCAO in rats treated with zinc in the presence or absence of L-NAME (Figure 1). The subacute administration of zinc in control group showed an increase of 79% ± 9% in zinc levels in the temporoparietal cortex on the third day, returning to basal levels on the fourth day of administration (Figure 1). The CCAO in rats treated with zinc caused an increase of 55% ± 5% at 4 h and of 43% ± 14% at 6 h postreperfusion, returning to the baseline at 24 h postreperfusion, maintaining this level in the 168 h (Figure 1). The L-NAME administration before the CCAO in rats treated with zinc caused several increases of zinc levels, from time 0 (CZn96h) of 112% ± 3%, decreasing at 4 h postreperfusion. A second increase of 70% ± 18% was observed at 36 h, a third increase of 103% ± 41% was observed at 72 h postreperfusion, and then decreasing by 68% ± 6% was observed at 96 h, returning again to the baseline at 168 h, after reperfusion (Figure 1).

**Figure 2:**
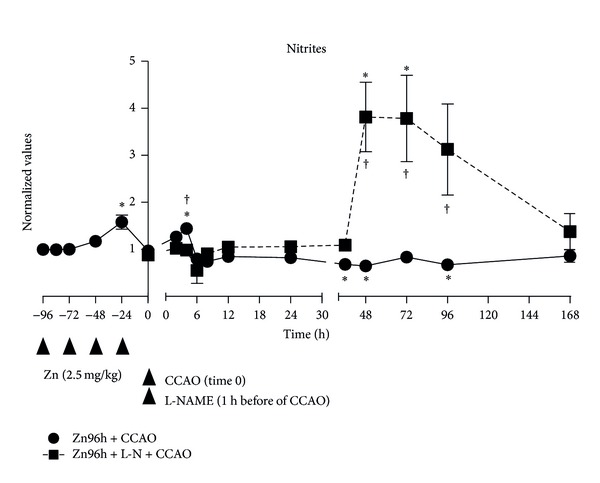
Subacute administration of zinc and L-NAME on nitrite levels during a cerebral hypoxia-ischemic process. The nitrite levels were assayed by the Griess method described elsewhere [18, 25]. Each value is the mean ± SE of 5 independent experiments made in triplicate. Zn96h + CCAO: preventive subacute administration of zinc (2.5 mg/kg each 24 hours during 4 days) and a common carotid-artery occlusion (CCAO) for 10 min. Zn96h + L-NM + CCAO: rats treated with zinc in presence of an inhibitor of nitric oxide synthase (L-NAME) one-hour before CCAO. *P < 0.05, an ANOVA test and a post-hoc Dunnet test to compare with control group, and ^†^ *P* < 0.05, unpaired Student's *t*-test to compare between groups.

 The subacute administration of zinc in the control group showed an increase of 58% ± 15% in the nitrite levels at the fourth dose of administration (Figure 2). The CCAO caused an increase of 45% ± 8% at 4 h postreperfusion in treated rats with zinc, decreasing at 8 h, but unchanged at the 168 h postreperfusion (Figure 2). However, the L-NAME administration in rats treated with zinc decreased nitrite levels during the firsts 36 h postreperfusion, but caused an increase of 282% ± 74% from 48 h postreperfusion, maintaining this level at 72 h, and then returned to the baseline at the 168 h postreperfusion (Figure 2).

 The systolic blood pressure of rats was measured before (P1) and after (P2) each treatment. The subacute administration of zinc in the absence of L-NAME did not change the systolic blood pressure before and after treatment, as compared to the controls without treatment. However, in rats treated with a subacute administration of zinc in the presence of L-NAME showed an increase of systolic blood pressure with Δ*P* of 25 (P1 was 130 ± 6 mm Hg and P2 was of 155 ± 3 mm Hg). 

To evaluate whether the intraperitoneal administration of zinc and L-NAME causes cell damage, several markers of damage (lipid peroxidation and caspase-3) were studied, and cellular death was analyzed by hematoxylin-eosin staining.

**Figure 3:**
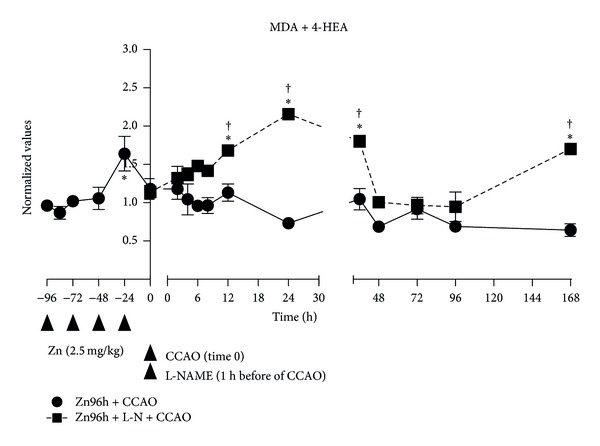
Subacute administration of zinc and L-NAME on lipoperoxidation levels during a cerebral hypoxia-ischemic process. Malonyldialdehyde (MDA) and 4-hydroxyalkanal (4-HEA) concentrations measured by using the method described elsewhere [18, 25] were used as biomarkers of lipoperoxidation. Each value represents the mean ± SE of 5 independent experiments made in triplicate. Zn96h + CCAO: preventive subacute administration of zinc (2.5 mg/kg each 24 hours for 4 days) and common carotid-artery occlusion (CCAO) for 10 min. Zn96h + L-NM + CCAO: rats treated with zinc in the presence of an inhibitor of nitric oxide synthase (L-NAME) one-hour before the CCAO. *P < 0.05, an ANOVA test and a post-hoc Dunnet test to compare with the control group, and ^†^ *P* < 0.05, unpaired Student's *t*-test to compare between groups.

The subacute administration of zinc caused an increase of 58% ± 15% in the MDA + HEA levels at the fourth dose, returning to baseline at the 24 h after the last administration (time 0; Figure 3). The CCAO in the rats treated with zinc did not change the MDA + HEA levels over 168 h after reperfusion (Figure 3). However, L-NAME administration before the CCAO in the rats treated with zinc showed an increase of 68% ± 6% at 12 h postreperfusion, with a maximum level of 116% ± 7% at 24 h, returning to baseline and then a second increase of 70% ± 5% was measured at 168 h postreperfusion (Figure 3).

**Figure 4:**
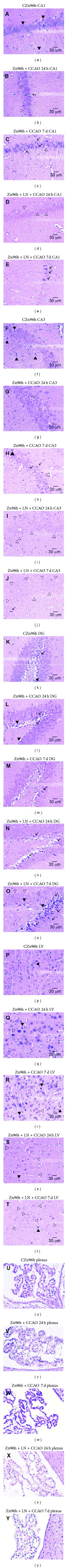
Hematoxylin-eosin staining in slides of the temporoparietal cortex and hippocampus in rats treated with zinc in the presence or absence of L-NAME. Paraffin-embedded tissue sections of 3 *μ*m were stained with hematoxylin and eosin. The Zn96h + CCAO: preventive subacute administration of zinc (2.5 mg/kg each 24 hours for 4 days) and a common carotid-artery occlusion (CCAO) for 10 min. Zn96h + L-NM + CCAO: rats treated with zinc in the presence of an inhibitor of nitric oxide synthase (L-NAME) one hour before a CCAO. CA1 (a–e), CA3 (f–j) regions and dentate gyrus (DG: (k–o)) of hippocampus and LV, layer V of cerebral cortex (p–t). Apoptosis cell (dark arrowhead), necrosis (clear arrowhead), and branched cells (arrow).

**Figure 5:**
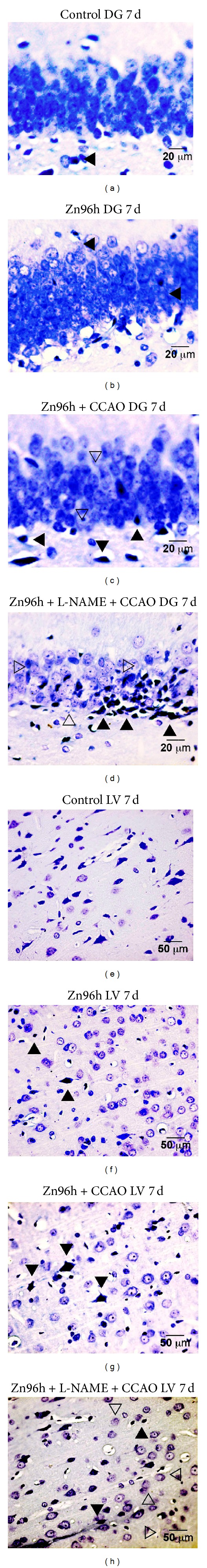
Immunohistochemistry against caspase-3 and Nissl counterstaining in slides of the hippocampus and temporoparietal cortex in zinc-treated rats in the presence or absence of L-NAME. The labels at the left side of the micrographs are cerebral regions. The immunostaining against cleaved caspase-3 is shown by the dark marks (dark arrowhead), and the Nissl stain appears in blue and pale cell (clear arrowhead). Zn96h + CCAO: preventive subacute administration of zinc (2.5 mg/kg each 24 hours for 4 days) and the common carotid-artery occlusion (CCAO) for 10 min. Zn96h + L-NAME + CCAO: rats treated with zinc in presence of an inhibitor of nitric oxide synthase (L-NAME) one-hour before the CCAO. DG: dentate gyrus of hippocampus; LV: layer V of cerebral cortex.

 The histopathological study qualitatively showed that zinc administration before the CCAO prevented cell death, whereas the L-NAME in rats treated with zinc caused morphological changes of necrosis and apoptosis (Figure 4) and higher cleaved caspase-3 IR cells from 24 h (data not shown), increasing at day 7 postreperfusion (Figure 5). 

 The histopathology studies showed that the subacute administration of zinc maintained the cellular structure of the hippocampus in the CA1, CA3, dentate gyrus, and the pyramidal neurons of the cerebral cortex at 24 h postreperfusion but caused changes in the morphology of the granular cells from the CA1 and CA3 at day 7 after reperfusion, showing elongated cells with ramifications, whereas the dentate gyrus showed a change at 24 h, returning to normal morphology at the day 7 postreperfusion. The choroid plexus did not show significant changes in its morphology, but basophilic nuclei were present in the cells (Figure 4). However, the administration of zinc and L-NAME decreased the color intensity in the nuclei of granular cells from CA1, CA3, and dentate gyrus at day 7 postreperfusion, suggesting the presence of cellular necrosis in the hippocampus and basophilic nucleus in the dentate gyrus, possibly due to apoptosis. The choroid plexus showed a decrease in the color intensity of the nucleus at day 7 after reperfusion (Figure 4). 

The subacute zinc administration caused a slight increase in the number of immunoreactivity (IR) cells against cleaved caspase-3 in the granular layer of the dentate gyrus (dg) and layer V (LV) of the temporoparietal cortex (arrowhead) at day 7 postreperfusion in control rats (Figure 5). However, the subacute administration of zinc also showed a slight increase of cleaved caspase-3 IR at the day 7 postreperfusion in both regions, whereas the L-NAME administration in the rats treated with zinc increased the caspase-3 IR cells in the granular layer of the dentate gyrus (dg) and layer five (LV) of the temporoparietal cortex at 24 h postreperfusion; however, it was more evident at day 7 postreperfusion. The other regions of hypothalamus also were affected (data not show). In addition, the Nissl staining (blue) showed a decrease in color intensity in the granular layer, it was more evident in the pyramidal cells of the LV of the temporoparietal cortex, with edema cells (arrow), and these results are indicative of cellular necrosis in the rats of the group Zn96h + L-NAME + CCAO (Figure 5).

## 4. Discussion

Subacute administration of zinc showed a cytoprotector effect leading to a decrease in the lipoperoxidation and immunoreactivity against cleaved caspase-3 by preventing an accumulation of zinc and increase of NO production in the late phase of a hypoxia-ischemia process. However, the coadministration of zinc and L-NAME (two inhibitors of NO production) showed a cytotoxic effect during a cerebral hypoxia ischemia in the rat, increasing the lipoperoxidation and cleaved caspase-3 IR cells from the early phase of a hypoxia-ischemia process, through the zinc accumulation in the early phase and an increase of zinc and the NO production in the late phase.

Prophylactic administration of zinc has a protector effect during hypoxia ischemia; similar results have been reported previously [2, 3, 5]. Other reports have shown neuroprotection when neuronal PC12 cells are preincubated with zinc salts, rather than coincubation [27] or the preventive subacute administration of zinc in rats [2].

Our work shows that a prophylactic subacute administration of zinc prevents the increase of NO caused by a CCAO in the late phase, maintaining the first increase of NO in the early phase of the hypoxia-ischemia process. The beneficial effect of NO in the early phase is related with the production of vasodilation [28] and metallothionein synthesis [29, 30]. In addition, NO acts as an antioxidant in the iron-mediated oxidative stress in rat hepatocytes, by an inhibition of lipoperoxidation [21]. It has been reported that NO participates in ischemic postconditioning process through the increase of NO by the eNOS pathway and attenuating an ischemia-reperfusion injury in liver [22]. Previous reports have shown that the antioxidant enzyme cooper-zinc superoxide dismutase (Cu-Zn SOD) decreases the oxidative stress produced by the NO derived from iNOS in diabetic animals [31] and cardiac embryopathy in maternal diabetes [32].

The stabilization of constitutive NOS (cNOS) by zinc [33, 34] could explain the increase in the NO level in the first hours during a hypoxia-ischemia process, which increases the cNOSs in the first hours of postreperfusion [35]. The zinc administration prevented the second increase of NO at 24 h by a CCAO, as was reported previously [18]. In addition, the zinc inhibits NF*κ*B and the synthesis of the iNOS protein [36], which has been reported at 12 h postreperfusion [35]. 

The beneficial effect of the zinc supplementation is to regulate the nitrosative stress, inducing antioxidant agents like glutathione [13, 37] and metallothionein [5], increasing the antioxidant capacity through Cu-Zn-SOD, storing the zinc in the intracellular compartment [38], and decreasing catalase and glutathione S-transferase activities [37].

Increased zinc levels found in the rats treated with zinc could cause a preconditioning, where the activation of zinc dependent of caspase-3 could be responsible for the cleavage of poly-ADP ribose polymerase (PARP), contributing to the decrease of the injury upon subsequent toxic exposure [39]. There is evidence that the extracellular zinc accumulation may be protective by preventing overactivation of the NMDA receptors. In addition, subtoxic accumulation intracellular of zinc may trigger a preconditioning effect, diminishing the susceptibility to a subsequent ischemia [39]. 

The subacute administration of zinc before a CCAO causes a decrease of the apoptosis at 24 h and in the day 7 after reperfusion, this has been reported previously by us [18] and others researchers in the hearts of diabetic mice [32]. The decrease of cleaved caspase-3 by zinc can be explained because zinc is able to directly inhibit the activity of caspase-3 [15, 16] and caspase-8, decreasing the cell death [17], since decrease proapoptotic proteins (Bax and Bak), or inhibits cytocrome c release [14].

It is known that NO participates in zinc accumulation and cell death [18, 40–43], and the administration alone of N-*ω*-nitro-L-arginine methyl ester (L-NAME) reduces the cerebral infarct [44], NOS activity, the edema [23], and cellular death at 24 h postreperfusion [18]. The administration of two inhibitors of NO production (zinc + L-NAME) causes an increase in systolic blood pressure and cell death in the cerebral cortex of the rat. Some reports found that the chronic inhibition of NO production causes hypertension [45] and decreases the plasma levels of zinc, causing small areas of myocardial coagulative necrosis [24].

The subacute administration of zinc and L-NAME caused an increase of zinc in the first and last hours, in which the increase of zinc could cause oxidative stress in the absence of NO in the first hours, where NO captures free radicals and prevents lipoperoxidation [46]. The increase of zinc in the late phase caused the increase of NO production and lipoperoxidation. The increase of zinc may trigger the generation of reactive oxygen species (produced by mitochondria, NADPH-oxidase, and other sources), which could cause the intracellular Zn mobilization through a voltage-dependent calcium channel [47] or ZIP transporter, the excessive release of presynaptic zinc (at micromolar concentration) [48, 49], and accumulation of zinc in a postsynaptic neuron [48] and in the mitochondria [50]. These mechanisms could produce damage to neurons (granular cells and pyramidal neurons) and glia, as reported elsewhere [8, 51]. Therefore, excessive zinc accumulation in the early and late phase could be cytotoxic, which is supported by an increase of apoptosis from the early phase and necrosis in the late phase. These findings are in agreement to the cytotoxic effect of zinc [52, 53], where the function of zinc is dependent on the concentration, space, and timing of the cellular response to injury.

## 5. Conclusions

 The treatment with two inhibitors of NO production (zinc and L-NAME) increased the cellular damage by necrosis and apoptosis in the hippocampus and layer V of cerebral cortex after a transient CCAO for 10 min through an increase of the nitrosative stress, zinc accumulation, and lipoperoxidation. This is opposite to that found with the subacute administration of only zinc before CCAO, which caused a cytoprotector effect by a decrease in the nitrosative stress and accumulation of zinc in the late phase. 

 These results provide evidence to consider the use of two therapeutic strategies that act on NOSs, to minimize damage during a cerebral hypoxic-ischemic process in patients who present a transient ischemia and are at high risk for a stroke or in patients with a disease cerebrovascular.
